# Effect of salt-alkali stress on seed germination of the halophyte *Halostachys caspica*

**DOI:** 10.1038/s41598-024-61737-5

**Published:** 2024-06-08

**Authors:** Rui Zhang, Huizhen Zhang, Lai Wang, Youling Zeng

**Affiliations:** Xinjiang Key Laboratory of Biological Resources and Genetic Engineering, College of Life Science and Technology, Xinjiang University, Ürümqi, 830017 China

**Keywords:** *Halostachys caspica*, Salt-alkali stress, Seed germination, Recovery germination, Relative salt damage, Biophysics, Plant sciences

## Abstract

The increasing global phenomenon of soil salinization has prompted heightened interest in the physiological ecology of plant salt and alkali tolerance. *Halostachys caspica *belonging to Amaranthaceae, an exceptionally salt-tolerant halophyte, is widely distributed in the arid and saline-alkali regions of Xinjiang, in Northwest China. Soil salinization and alkalinization frequently co-occur in nature, but very few studies focus on the interactive effects of various salt and alkali stress on plants. In this study, the impacts on the *H. caspica* seed germination, germination recovery and seedling growth were investigated under the salt and alkali stress. The results showed that the seed germination percentage was not significantly reduced at low salinity at pH 5.30–9.60, but decreased with elevated salt concentration and pH. Immediately after, salt was removed, ungerminated seeds under high salt concentration treatment exhibited a higher recovery germination percentage, indicating seed germination of *H. caspica* was inhibited under the condition of high salt-alkali stress. Stepwise regression analysis indicated that, at the same salt concentrations, alkaline salts exerted a more severe inhibition on seed germination, compared to neutral salts. The detrimental effects of salinity or high pH alone were less serious than their combination. Salt concentration, pH value, and their interactions had inhibitory effects on seed germination, with salinity being the decisive factor, while pH played a secondary role in salt-alkali mixed stress.

## Introduction

Soil salinization has become a worldwide ecological problem, and its degree and area are increasing year by year, restricting the global agricultural development^1–3^. It is estimated that approximately one-third of arable land throughout the world is affected by natural and secondary salinity to varying degrees^4,5^. Xinjiang encompasses a salt-alkali soil area of 2181.4 × 10^4^ hm^2^, representing 22.01% of China’s total area with the broadest distribution of soil salinization in China^6,7^. Therefore, soil salinization is a widespread abiotic stress source and has become a major constraint and obstacle to agricultural crop production and sustainable development^8^.

Salt-alkali stress is the second largest abiotic stress factor that hinders the normal growth and development of crops after drought stress^9^. Salt stress is mainly composed of neutral salts, such as NaCl and Na_2_SO_4_, pH value is between 7 and 8. Alkali stress is mainly generated by alkaline salts, such as NaHCO_3_ and Na_2_CO_3_ with high pH (more than 8.5)^10,11^. In fact, soil salinization and alkalinization frequently co-occur in nature. Most of the salinized soils in Xinjiang are mainly composed of NaCl, Na_2_SO_4_, Na_2_CO_3_ and NaHCO_3_. So the components of the salt-alkali soils in fields are complex, some salt-alkali soils have high salinity, but low pH, while some have low salinity, but high pH. Salt-alkali stress will damage plant cell structure, the stability of cell osmotic pressure and disturb plant nutrition absorption^12,13^.

Seed germination is the most critical phase in plant life cycle and sensitive to salt-alkaline stress^14–17^. Tolerance to salinity at the seed germination stage can evaluate the ability of the plant to resist salinity. High germination percentage in hypersaline environments together with the ability to recover germination after the remove of salinity are indicators of salt tolerance^18^. Low salt concentrations promote seed germination, while high salinity significantly inhibits seed germination. For example, the seed germination of these halophytes (*Haloxylon ammodendron*^19^, *Halostachys caspica*^20^, *Centaurea ragusina*^21^) was higher under low concentrations of neutral salts (Na_2_SO_4_ or NaCl). Conversely, the seed germination of the annual halophyte *Cakile maritima* under 200 mmol/L NaCl was severely inhibited and delayed, resulting in shortened seedling length^22^. However, numerous studies have demonstrated that alkali salt stress is more detrimental to plants than neutral salt stress^23–26^. For instance, when pepper seeds were exposed to neutral salt and alkali salt at the same concentration, alkali salt exhibited a stronger inhibitory effect on pepper seed germination^27^. Plants are exposed not only to the stress of individual neutral or alkali salt, but also to a complex environment of salt-alkali interaction. The interactive impact of salt-alkali stress to plants is greater than that of individual salt or alkali stress, with alkali stress causing a greater degree of harm than salt stress^24,26,28^. Studies have indicated that varying salt stress concentrations have diverse effects on the growth and physiological characteristics of halophytes, appropriate salt-alkali concentration is beneficial for seed germination^29^.

*Halostachys caspica* (Bieb.) C. A. Mey, a perennial halophytic shrub of the Amaranthaceae family, possesses highly succulent assimilating branches capable of reducing soil salt content and increasing soil organic matter. This plant exhibits strong adaptability to saline soils, being one of the primary halophytes in the arid desert region of Xinjiang. It has multiple abiotic tolerances, such as drought tolerance, salt-alkali tolerance, wind erosion resistance, and sand burial tolerance. Consequently, it plays a crucial role in preventing land desertification and also serves as an important medicinal and forage plant^30^. Currently, research concerning *H. caspica* primarily focued on seed germination^31^, analysis of medicinal components^32–34^, evaluation of feeding value^35^, and function identification of stress response genes and mechanisms explanation underlying single salt stress^36–38^. This study explored the effect of various types of neutral salt, alkali salt, or salt-alkaline mixed stress on the seed germination of *H. caspica*. Our purpose was to investigate the tolerance and adaptability of *H. caspica* under salt-alkali stress during seed germination, providing a theoretical basis for its cultivation and management, and laying the foundation for further research on the salt tolerance mechanism and utilization of saline-alkali land.

## Materials and methods

### Seed collection

The seeds of *H. caspica* were collected from plants growing wildly in the extremely salt-alkali and semi-desert regions located at the edge of Gurbantunggut Desert in Xinjiang, Northwest of China.

Collection of plant material comply with relevant institutional, national, and international guidelines and legislation.

### Seed surface disinfection

For the germination experiments, healthy and uniform seeds was selected and subjected to 1% sodium hypochlorite for 30 min. Subsequently, the seeds were rinsed three times with distilled water, and their surface moisture was dried using clean filter paper.

### Salt and alkali solution design

Salt stress was designed with single neutral salt NaCl (pH 5.45 ± 0.15) and mixed neutral salts (NaCl and Na_2_SO_4_, at a 1:1 molar ratio, pH 6.15 ± 0.15). Alkali salt stress was applied with single alkali salt NaHCO_3_ (pH 8.55 ± 0.35) and mixed alkali salts (NaHCO_3_ and Na_2_CO_3_, at a 1:1 molar ratio, pH 9.6 ± 0.10). Mixed salt-alkali stress was designed with NaCl and NaHCO_3_, at a 1:1, 1:3 and 3:1 molar ratio, (pH 8.25 ± 0.35). All salt solution treatments were initially configured as 1 mol/L stock solutions and then diluted to different concentrations (50, 100, 200, 300, 400, 500, 600 mmol/L) for seed germination. The accurate pH values were shown in Table 1.

**Table 1:** pH of solutions with different salt-alkali types.

Different treatments	pH of solutions with different concentrations								
Salt-alkali solutions	Stock solutions 1 mol/L	50 mmol/L	100 mmol/L	200 mmol/L	300 mmol/L	400 mmol/L	500 mmol/L	600 mmol/L	pH
NaCl	5.5	5.6	5.6	5.5	5.5	5.5	5.4	5.3	pH 5.45 ± 0.15
NaCl: Na_2_SO_4_	6.2	6.3	6.3	6.2	6.2	6.2	6.1	6.0	pH 6.15 ± 0.15
NaHCO_3_	8.7	8.4	8.3	8.6	8.5	8.7	8.7	8.9	pH 8.55 ± 0.35
NaHCO_3_: Na_2_CO_3_	9.7	9.5	9.5	9.5	9.6	9.6	9.6	9.7	pH 9.60 ± 0.10
NaCl: NaHCO_3_ = 1:1	8.3	8.2	8.1	8.4	8.3	8.4	8.6	8.4	pH 8.30 ± 0.30
NaCl: NaHCO_3_ = 1:3	8.6	8.3	8.3	8.4	8.5	8.6	8.5	8.6	pH 8.45 ± 0.15
NaCl: NaHCO_3_ = 3:1	8.0	8.0	8.0	7.9	8.2	8.2	8.2	8.3	pH 8.10 ± 0.20

### Salt stress treatment

5 mL of the respective salt solution was added in each 90 mm petri dish with two layers of filter paper, and then sterilized seeds were placed on the moistened filter paper. Germination occurred under a 16 h/8 h (light/dark) cycle at temperatures of 25 ℃/15 ℃, with relative humidity at 60% and light intensity at 56 μmol/(m^2^·s). Seed germination was recorded when radicle emergence reached 2 mm. The corresponding salt solution was replaced every 2 days to maintain consistent solution concentrations. Germinated seeds were counted every 24 h, continuously observed for 13 days, and the final germination percentage was calculated. Each treatment was included three replicates, with 40 seeds per petri dish, and the distilled water treatment group was served as the control.

### Recovery treatment

The ungerminated seeds subjected to salt stress treatment were washed with distilled water three times before being transferred to a culture of distilled water. The number of seed germination recovery was recorded every 24 h, and continuous observation was conducted over a period of 7 days.

### Evaluation of salt and alkali tolerance

The evaluation of salt and alkali tolerance on the stage of seed germination was followed this criteria outlined in Wang et al.^39^. The germination percentages resulting from the application of NaCl, NaHCO_3_, and mixed stress treatments were served as the dependent variable (y), while the concentrations of the respective salt stress treatments were served as the independent variable (x) in establishing a regression equation. The salt-alkali solution concentration corresponding to the germination percentages of *H. caspica* at 75%, 50%, and 25% were defined as the moderate, threshold, and limit values for the salt-alkali tolerance of seed germination.

### Data analysis

The parameters were measured under stress conditions, including germination percentage (G_s_), recovery germination percentage (G_r_), total germination percentage (G_t_) and relative salt damage percentage (RSD).

$${\text{G}}_{{\text{s}}} \left( \% \right) \, = \, \left( {{\text{A}}/{\text{C}}} \right) \, *{1}00$$

$${\text{G}}_{{\text{r}}} \left( \% \right) \, = \, \left[ {{\text{B}}/\left( {{\text{C}} - {\text{A}}} \right)} \right] \, *{1}00$$

$${\text{G}}_{{\text{t}}} \left( \% \right) \, = \, \left[ {\left( {{\text{A}} + {\text{B}}} \right)/{\text{C}}} \right] \, *{1}00$$

$${\text{RSD }}\left( \% \right) \, = \, [({\text{G}}_{{{\text{ck}}}} - {\text{G}}_{{\text{s}}} )/{\text{ G}}_{{{\text{ck}}}} ] \, *{1}00$$

A: Number of germinated seeds under stress.

B: Number of germinated seeds being transferred to distilled water after the removal of stress.

C: Total number of seeds per treatment.

Germination percentage, total germination percentage, and recovery germination percentage were transformed into arcsines prior before the data were analyzed. Each treatment was consisted of three replicates, and the data were presented as mean ± standard error (SE).

One-way analysis of variance (ANOVA) was conducted using SPSS 20.0 (SPSS Inc., Chicago, IL, USA) to assess significance. Additionally, two-way ANOVA was employed to examine the individual and interactive effects of salt and pH on the variables. The means among the treatment were compared using post hoc Tukey’s honestly significant difference (HSD) test. Differences were considered significant at *p *≤ 0.05. Graphs were generated using GraphPad Prism version 5.0 (GraphPad software, San Diego, CA, USA), and regression equations were computed using Excel 2019.

## Results

### Effect of neutral and alkaline salts on seed germination of *H. caspica*

**Figure 1:**
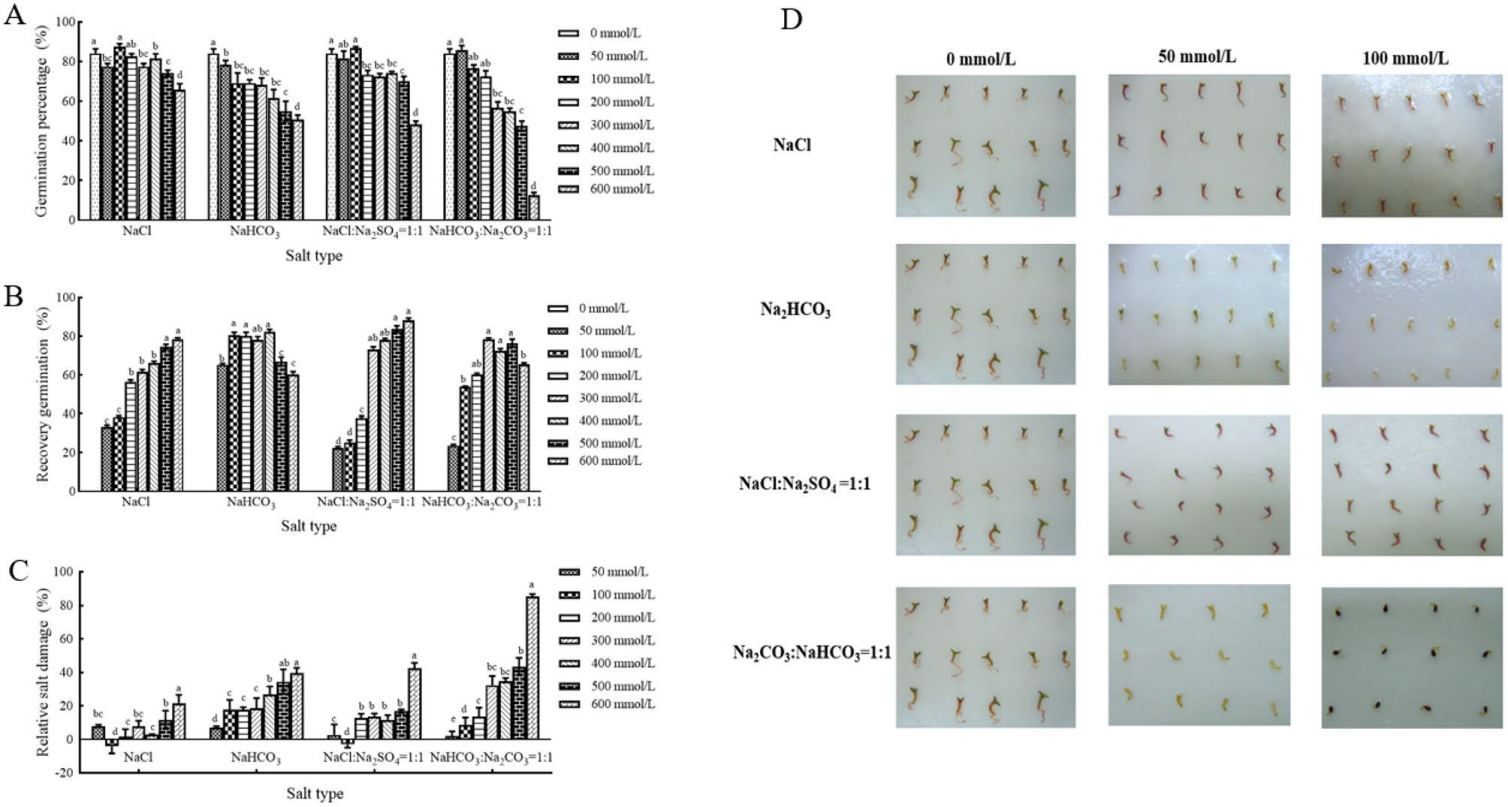
Seed germination of *H. caspica* under neutral or alkaline salt stress. The germination percentage (**A**), recovery germination percentage (**B**), relative salt damage percentage (**C**), germinated phenotypes (**D**) of *H. caspica* seeds under different salt stress. The same letters in the figure represented no significant differences (*p* > 0.05), and different letters indicated significant differences (*p* < 0.05).

The type and concentration of salt, as well as their interaction, significantly influence the germination percentage of *H. caspica* seeds (*p *< 0.05). High germination percentages were observed in the control (the treatment of distilled water), and the germination percentage was decreased with increasing salinity. From Fig. 1A illustrated that low concentrations of neutral salt (NaCl) or alkaline salt (NaHCO_3_) had almost minimal inhibitory effect on the germination of *H. caspica* seeds. However, with increasing salt concentration, the germination significantly was reduced. Under the same salt concentration, the seed germination was significantly decreased under alkaline salt stress, compared with neutral salt treatment. Additionally, mixed neutral or mixed alkaline salt inhibited seeds germination more severely than single neutral or alkaline salt.

Ungerminated seeds subjected to neutral or alkaline salt stress were transferred to distilled water for rehydration testing, and the germination percentage of the seeds was calculated. Following the removal of salt stress, some *H. caspica* seeds resumed germination rapidly. As shown in Fig. 1B, ungerminated seeds treated under high salt concentrations exhibited the highest recovery germination percentage. This suggested that under high salt stress, seed could still remain energetic with seed germination being inhibited. When the pressure was removed, seeds could promptly recover germination.

The relative salt damage percentage is a pivotal indicator for evaluating salt tolerance during seed germination stage. The results indicated that the RSD of seeds showed an increasing trend with deepening salt concentration. Under equivalent salt concentration stress, the highest RSD occurred under mixed alkaline salt (NaHCO_3_ and Na_2_CO_3_) conditions. Furthermore, it was observed that at a concentration of 100 mmol/L, a negative RSD was yielded with both single neutral salt (NaCl) and mixed neutral salt (NaCl and Na_2_SO_4_) treatment group, suggesting a promotive effect on the *H. caspica* seed germination (Fig. 1C).

The external morphology and growth status are the most intuitive manifestation of the degree of salt-alkali damage to plants. The results indicated that, in comparison to the control group, there was no significant difference in phenotype between the single and mixed neutral salt treatment groups. However, significant differences in phenotype were observed between the single and mixed alkaline salt treatment groups and the control group. Additionally, with increasing alkaline salt concentration, the root length and aboveground biomass of *H. caspica* were significantly decreased (Fig. 1D).

In order to further elucidate the tolerance levels of the *H. caspica* seed germination and germinated seedling growth under neutral salt, alkaline salt, and mixed salt-alkali stress, a curve regression analysis was conducted between the seed germination percentage and salt concentration. The results showed a strong linear relationship between germination percentage and salt concentration, exhibiting an extremely significant negative correlation (Fig. S1). Utilizing the established optimal function equation, moderate, threshold, and limit values of salt concentration during the germination stage of the *H. caspica* seeds were determined, revealing the tolerance order to the four treatments for this species’ germination, as NaCl > NaCl: Na_2_SO_4_ = 1:1 > NaHCO_3_ > NaHCO_3_: Na_2_CO_3_ = 1:1 (Tables 2 and S3).

**Table 2:** Regression analysis of neutral and alkaline salt concentration with germination percentage.

Salt types	Regression equation	R^2^	Moderate value (mmol/L)	Threshold (mmol/L)	Limit (mmol/L)
NaCl	y = − 0.0236x + 85.207	0.5856	432.5000	1491.8220	2551.1440
NaHCO_3_	y = − 0.049x + 80.249	0.9253	107.1224	617.3265	1127.5306
NaCl: Na_2_SO_4_ = 1:1	y = − 0.0477x + 86.664	0.7605	244.5283	768.6373	1292.7463
NaHCO_3_: Na_2_CO_3_ = 1:1	y = − 0.1052x + 89.622	0.8989	138.9924	376.6350	614.2776

### Effect of salt-alkali mixed stress on seed germination of *H. caspica*

**Figure 2:**
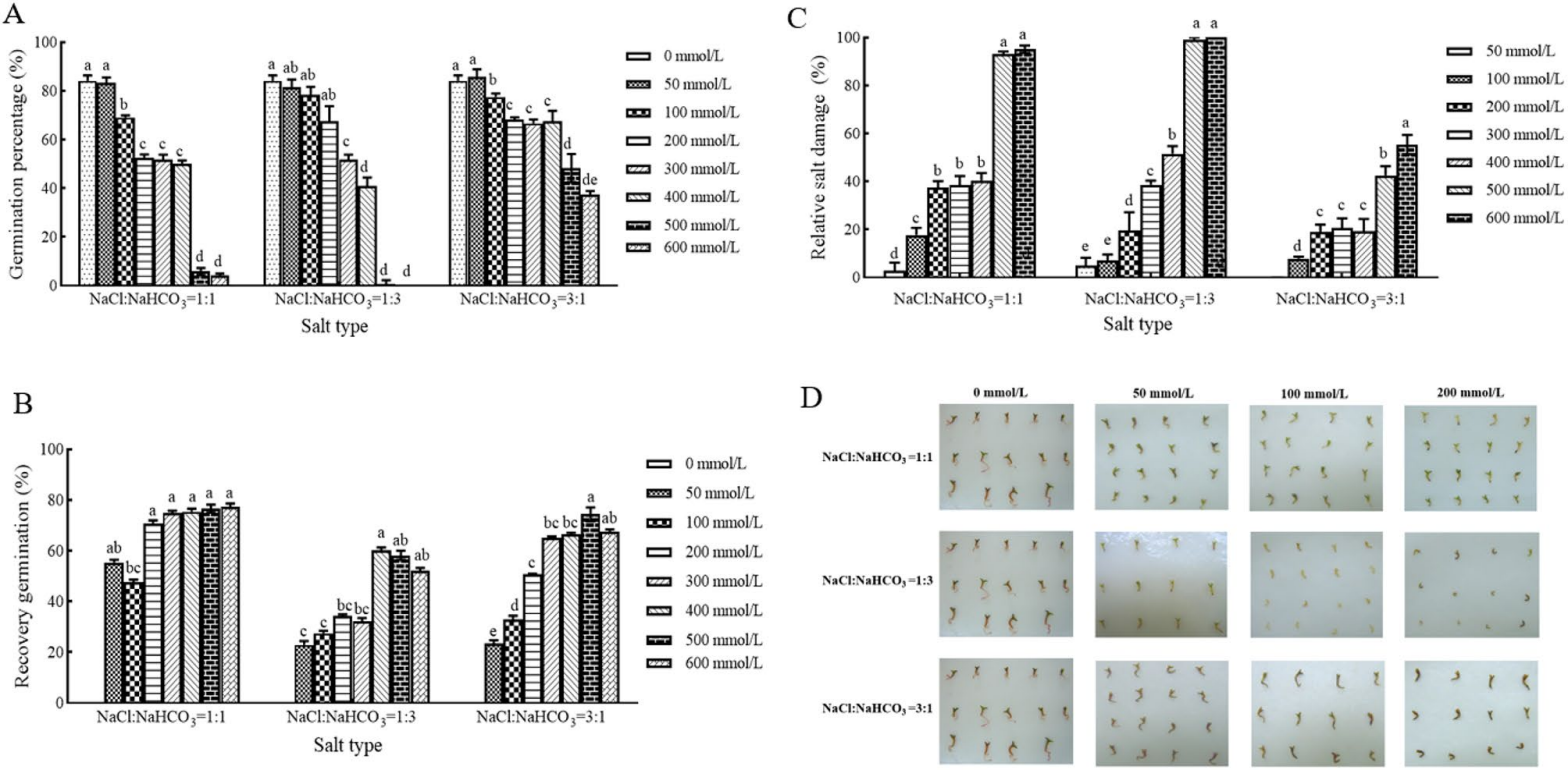
Seed germination of *H. caspica* under salt-alkali mixed stress. The germination percentage (**A**), recovery germination percentage (**B**), relative salt damage percentage (**C**), germinated phenotypes (**D**) of *H. caspica* seeds under mixed salt-alkali stress. The same letters in the figure represented no significant differences (*p* > 0.05), and different letters presented significant differences (*p* < 0.05).

Mixed salt-alkali stress differs from individual salt or alkali stress. The main characteristic of salt-alkali mixed stress is the mutual enhancement, compared to single salt and alkali stress. As illustrated in Fig. 2A, mixed salt-alkali stress exerted a strong inhibitory effect on the germination of *H. caspica* seeds. At a salt concentration of 500 mmol/L NaCl: NaHCO3 = 1:1, the seed germination percentage was only 5.83%. Moreover, with the increase in alkaline salt content in mixed salt-alkali stress, the inhibitory effect on the germination of *H. caspica* seeds was intensified.

The germination recovery experiment revealed that ungerminated seeds initiated germination within 2 days after transferred to distilled water, with the peak recovery period from the 3rd to the 5th day. Ungerminated *H. caspica* seeds exposed to high concentrations (  ≥ 400 mmol/L) of mixed salt-alkali stress yielded a relatively high germination recovery percentage (Fig. 2B). There was on significant change observed in the total germination percentage being up approximately 80% under the mixed salt-alkali treatment with salt concentration (≤ 400 mmol/L) and pH 7.9-8.6 (Table S2).

The mixed salt-alkali stress significantly damaged the seeds of *H. caspica*. At a mixed salt-alkali concentration of 500 mmol/L, the relative  salt damage percentage reached as high as 90%. Moreover, an increase in the neutral salt (NaCl) content within the mixed salt-alkali solution led to a decrease on the RSD, while an increase in the alkaline salt content intensified the relative salt damage degree (Fig. 2C, Table S4). Consequently, the tolerance order on the *H. caspica* seed germination under three different treatments was as follows: NaCl: NaHCO_3_ = 3:1 > NaCl: NaHCO_3_ = 1:1 > NaCl: NaHCO_3_ = 1:3 (Table 3, Fig. S2). These phenotypic results were also consistent with these findings (Fig. 2D).

**Table 3:** Regression analysis of salt-alkali mixed concentration with germination percentage.

Salt type	Regression equation	R^2^	Moderate value (mmol/L)	Threshold (mmol/L)	Limit (mmol/L)
NaCl:NaHCO_3_ = 1:1	y = − 0.1351x + 86.418	0.9112	84.5150	269.5633	639.6595
NaCl:NaHCO_3_ = 1:3	y = − 0.1529x + 91.720	0.9451	109.3525	272.8581	599.8692
NaCl:NaHCO_3_ = 3:1	y = − 0.0736x + 86.757	0.9152	159.7418	499.4158	1178.7636

In summary, these findings suggested that the inhibitory effect of mixed salt-alkali treatment on *H. caspica* seed germination was primarily attributed to alkaline salts. Moreover, the greater the proportion of alkaline salts was, the more dramatical the inhibitory effect on seed germination was.

The results of the two-factor variance analysis indicated that salt concentration (F = 112.135, *p* < 0.01) predominantly contributed to the inhibition of seed germination in *H. caspica*, while pH value (F = 76.713, *p* < 0.01) also played a dominant role in this inhibition. Additionally, the interaction between salt concentration and pH value also significantly influenced seed germination (Table 4).

**Table 4:** Two-way ANOVA of effects of salt, pH, and their interaction on seed germination of *H. caspica.*

Variance source	Germination rate
Salt concentration	112.135**
pH	76.713**
Salt concentration × pH	13.122*

*, 0.05; **, 0.01.

## Discussion

### *H. caspica* has strong salt and alkali tolerance

Salt-alkali stress on plants leads to a series of comprehensive effects, mainly affecting plant growth and development by reducing water absorption capacity, disrupting ion balance, inducing hypertonic stress, and damaging the photosynthetic system^14,40^. Plant salt tolerance varies with different developmental stages, and especially, on the seed germination stage, plant is the most sensitive to salt -alkali stress^15^. Numerous studies had reported that under salt alkali stress, the germination time of seeds was delayed, the germination percentage was decreased, and some seeds even became completely inactive and die^11^. In this study, it was observed that some *H. caspica* seeds germinated normally even under high neutral and alkaline salt concentrations, indicating *H. capsica* seeds exhibit strong adaptability to high salt-alkali environments.

Ungerminated seeds of many halophytes subjected to high salinity can recover germination, when transfered to distilled water^16,41^. However, for some species, seed germination remains permanently inhibited by high salinity^19,21^. In our study, although salt stress inhibited the germination of *H. capsica* seeds to varying degrees, some seeds resumed their germination after the removal of stress. In a high pH environment with salt concentration (≤ 400 mmol/L), the total germination percentage of *H. capsica* seeds was more than 80%, while in a high pH with high salt concentration (> 400 mmol/L), the total germination percentage was gradually decreased (Table S2), suggested *H. caspica* at the seed germination stage had strong tolerance to both salt and alkali stress, but high alkali stress caused certain damage for *H. caspica* seeds indeed.

### Salt concentration and pH value are the main factors affecting the germination of *H. caspica*

Seed germination is a complex physiological process highly susceptible to environmental stress. The rate of seed germination is contingent upon the speed of water absorption. Salinity decelerated water absorption, impeding cell membrane repair during the absorption process and exacerbating membrane structure damage, thereby causing solute exudation from the seeds^42^. As salinity increased, the extent of seed damage became  intensively, ultimately impacting seed germination^25,26,29^.

A close relationship between the germination percentage of *H. caspica* seeds and the salt concentration was observed. The inhibitory effect of low salt concentration on seed germination was not significant. However, when the salt concentration exceeded 200 mmol/L, the inhibition of seed germination was deepened (Fig. 1A, 2A). Under the same pH condition, the germination percentage of *H. caspica* seeds was decreased and the relative salt damage percentage was increased with increasing salt concentration. This phenomenon may be attributed to the salt solution concentration, as higher osmotic pressure led to greater separation of the cytoplasmic wall, suppressing the seed’s swelling. Or the toxic effect of high salt ion concentrations inhibited enzyme activity, and affected metabolic activity within the cell, resulting in a decreased in all germination indicators^13,15,22^.

Numerous experimental data indicated that a high pH value was the key to restricting the growth and development of plants in alkaline conditions^10,43^. Soil alkaline pH stress can modify apoplastic pH and influence the intracellular pH environment^44–46^, thereby regulating numerous metabolic and physiological processes, including photosynthesis^47^, ionic homeostasis^48,49^, membrane transport^50,51^ and reactive oxygen species (ROS) balance^52,53^. Alkaline salts, containing HCO_3_^−^ and CO_3_^2−^ can elevate the pH of solutions. Low concentrations of alkaline salts may not affect seed water absorption, but as the concentration of alkaline salts was increased, the seed germination percentage was sharply decreased, indicating that high pH is one of the limiting factors for seed germination^54–56^.

In this study, under the same salt concentrations, the germination percentage of *H. caspica* seeds was markedly decreased, as the pH of the salt solution was increased. Meanwhile, it was found that the impact of high pH on the inhibition of seed germination could intensify with increasing salinity (Fig. 2A). This phenomenon may be attributed to the gradual increase in the proportion of alkaline salts during salt-alkali stress, resulting in elevated pH. High pH in solutes inhibited of key enzyme activities for germination within the seeds, led to nutrient deficiency within the cells, and ultimately impeded seed germination^24,27,39^.

The results of the two-factor variance analysis in this study showed that under salt stress treatment, the germination indicators of *H. caspica* seeds were jointly affected by salt concentration, pH, and the interaction between the both. The degree of influence was the strongest with salt concentration, followed by pH, and the interaction between the both was the smallest, which was consistent with previous research results on *Mentha sachalinensis*^29^, *Bassia dasyphylla*^57^. Under complex salt-alkali environments, seed germination was not only affected by salt stress, but also by the environmental pH and their interaction, with varying degrees of influence depending on plant species and varieties^28^.

### The inhibitory effect of alkaline salt stress on *H. caspica* is stronger than that of neutral salt stress

Salt and alkali stresses imposes varying degrees of damage on plants, with the tolerance difference among different plant species. At present, numerous studies had demonstrated that alkaline salt stress exerted a greater inhibitory effect on plant growth, compared to neutral salt stress^57^.

Halophyte seeds could maintain viability even after prolonged exposure to salt stress and can germinate under favorable conditions^16^. Additionally, research indicates that low concentrations of neutral salt solutions can enhance the germination of some plant seeds, such as *Haloxylon ammodendron*^19^ and *Centaurea ragusina*^21^. Our experiment also revealed that the low concentration treatments (100 mmol/L) with neutral salt NaCl or a mixture neutral salts (NaCl and Na_2_SO_4_) improved the germination percentage of *H. caspica,* compared to the control (CK), with the relative salt damage percentage of *H. caspica* seed germination being negative, indicating a promotion of *H. caspica* seed germination at this concentration. Single alkali salt NaHCO_3_ or mixed alkali salt (NaHCO_3_: Na_2_CO_3_) significantly inhibited seed germination. For example, in high salt concentration (600 mmol/L), seed germination percentage and recovery germination percentage were 12.5% and 65.53% in the treatment of mixed alkali salts, while 48.33% and 88.05% in the condition of mixed neutral salts (NaCl and Na_2_SO_4_). These results were consisted with previous research reports^23,24,26^.

Therefore, alkaline salt stress had a stronger inhibitory effect on the *H. caspica* seed germination than neutral salt stress.

### The inhibitory effect of mixed salt-alkali treatment on *H. caspica* seed germination is stronger than that of single salt or single alkaline salt treatment

Research demonstrated that mixed salt-alkali stress significantly differs from individual salt or alkali stress, presenting a more intricate scenario than either stress alone^58,59^. Notably, the significant feature of salt-alkali mixed stress is the mutual enhancement of salt and alkali stress to plants. In nature, high salinity and elevated pH often coincide, and their synergistic interaction may exert a greater impact on plant growth and development than either stress alone. Mixed salt-alkali stress led to a more severe ion imbalance, reduced osmoregulation ability, inhibited the antioxidant system, and resulted in more severe inhibition of plant growth^60–63^.

This experiment also showed that the mixed salt-alkali treatment group exhibited greater inhibition on the germination percentage of *H. caspica* seeds, compared to the groups subjected to single salt or single alkali treatment. Such as, in high salt concentration (600 mmol/L), seed germination percentage was 4.17% in the treatment of mixed salt-alkali stress (NaCl:NaHCO_3_ = 1:1), while it was 65.83% and 50.03% in the condition of single neutral salt (NaCl) and single alkali salt (NaHCO_3_). Salt-alkali stress commonly exerted negative impacts on plant growth and development primarily through ionic osmosis and high pH stress, rendering it more detrimental than salt or alkali stress alone^64,65^.

## Conclusions

The present experiment aimed to assess the impact of neutral salts, alkaline salt, and mixed salt-alkali stress on the germination and recovery of *H. caspica* seeds. Findings revealed the following results: (1) As salt concentration and pH value increased, the germination percentage of *H. caspica* seeds exhibited a decreasing trend. Furthermore, under the same salt concentration but varying pH treatments, the germination percentage of *H. caspica* seeds reduced, as pH increased. (2) Low concentrations of neutral salts had a promotional effect on the germination of *H. caspica* seeds. (3) Salt-alkali mixed stress exerted a stronger inhibitory effect on *H. caspica* seeds than single neutral or alkaline salt stress. (4) Following transferring ungerminated seeds to distilled water for rehydration, the percentage of germination recovery increased with the treatment of higher salt concentrations, while no significant difference was observed in the overall germination percentage. (5) Salt concentration, pH value, and their interactions had inhibitory effects on the germination of *H. caspica* seeds, with salt concentration being the decisive dominant factor, followed by the influence of pH value on seed germination.

All in all, our study indicated that *H. caspica* has strong salt-alkali tolerance at least during the seed germination stage. The halophytes *H. caspica* can survive and produce a considerable biomass in severely salt-alkali soils. It is a valuable resources for crop stress tolerant improvement and development and utilization of saline alkali land.

### Supplementary Information

Supplementary Information.

## Data Availability

The authors confirm that the data supporting the findings of this study are available within the article and its supplementary materials.
