# Supplementary material for: Effect of salt-alkali stress on seed germination of the halophyte Halostachys caspica
Source: Sci Rep. 2024 Jun 8;14:13199. doi: 10.1038/s41598-024-61737-5 (PMC11162456; doi:10.1038/s41598-024-61737-5)
Supplement: Supplementary file 1 — Supplementary Information. [file 41598_2024_61737_MOESM1_ESM.docx]

**Effect of Salt-alkali Stress on Seed Germination of the Halophyte *Halostachys caspica***

**Rui Zhang, Huizhen Zhang, Lai Wang, Youling Zeng***

**Supplementary Data**

**Figure S1**

**A B**

**
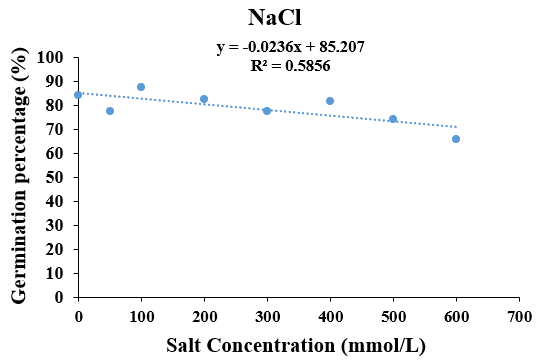

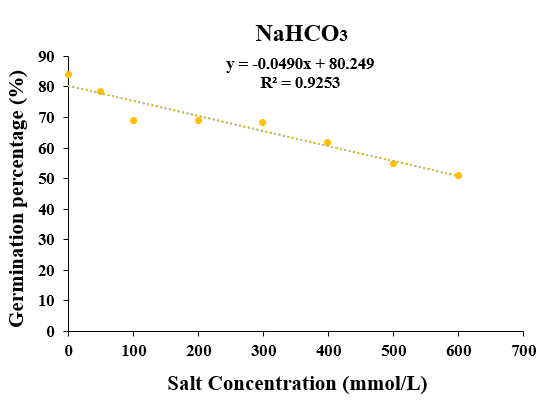
**

**C D**

**
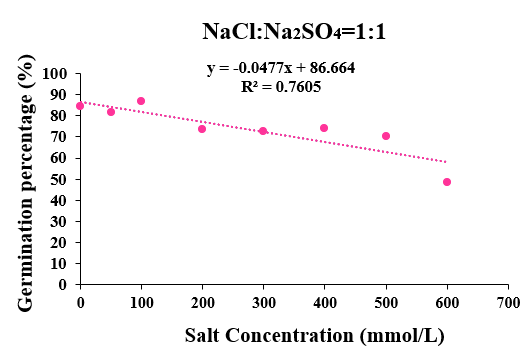

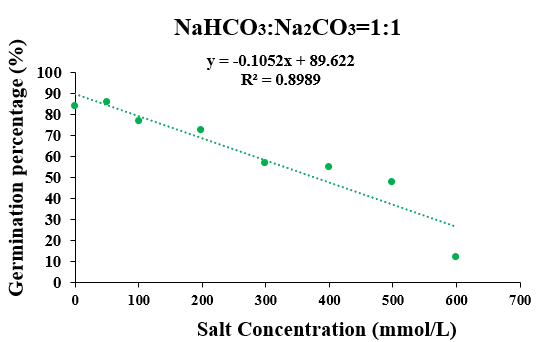
**

**Fig. S1** Linear regression equation on the *H. caspica* seed germination under various treatments of single neutral salt, single alkali salt, and mixed neutral salt, mixed alkali salt. (A) NaCl; (B) NaHCO_3_; (C) NaCl: Na_2_SO_4_=1:1; (D) NaHCO_3_: Na_2_CO_3_=1:1.

**Figure S2**

**A B**

**
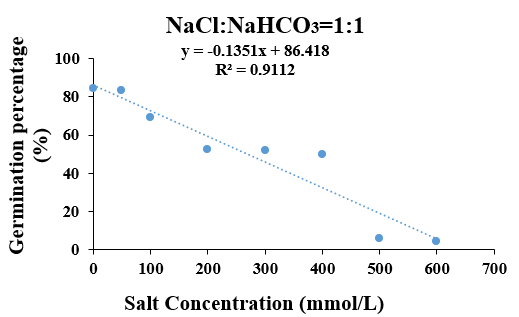

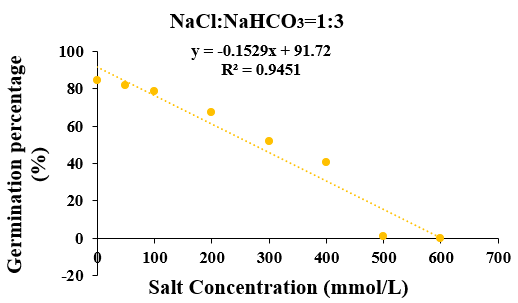
**

**C**

**
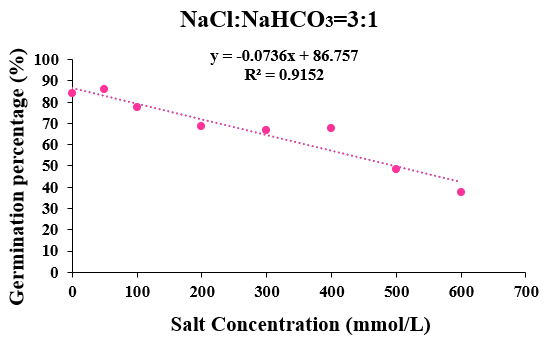
**

**Fig. S2** Linear regression equation on the *H. caspica* seed germination under the treatments of the mixed salt-alkali stress with different proportion. (A) NaCl:NaHCO_3_=1:1; (B) NaCl:NaHCO_3_=1:3; (C) NaCl:NaHCO_3_=3:1.

**Supplementary Table S1.** Effect of different salt-alkali treatments on the seed germination of *H. caspica*

| **Different treatment** | **Salt concentration**  **(mmol/L)** | **G_s_ (A/C)*100** | **G_r_[B/(C-A)]*100** | **G_t_[(A+B)/C]*100** |
| --- | --- | --- | --- | --- |
|  | CK | 84.17±1.25^a^ | 0.00±0.00^d^ | 84.17±1.25^b^ |
|  | 50 | 77.5±0.82^b^ | 33.33±1.69^c^ | 86.66±2.53^ab^ |
|  | 100 | 87.5±0.82^a^ | 38.33±0.82^c^ | 90.23±1.23^a^ |
| NaCl | 200 | 82.50±0.82^ab^ | 57.15±0.82^b^ | 84.37±1.25^ab^ |
|  | 300 | 77.5±0.81^bc^ | 62.96±1.26^b^ | 86.54±2.10^ab^ |
|  | 400 | 81.67±1.25^ab^ | 66.67±1.25^b^ | 91.16±1.21^a^ |
|  | 500 | 74.17±0.47^c^ | 74.19±0.47^a^ | 83.01±2.35^ab^ |
|  | 600 | 65.83±1.70^d^ | 78.05±0.94^a^ | 82.65±1.11^ab^ |
|  |  |  |  |  |
|  | CK | 84.17±1.25^a^ | 0.00±0.00^d^ | 84.17±1.25^a^ |
|  | 50 | 78.33±1.54^b^ | 65.38±1.25^c^ | 87.36±2.35^a^ |
|  | 100 | 70.12±3.51^bc^ | 81.08±3.27^a^ | 85.12±3.23^a^ |
| NaHCO_3_ | 200 | 69.17±1.15^bc^ | 80.2±0.82^ab^ | 83.24±1.21^a^ |
|  | 300 | 68.33±2.31^bc^ | 78.96±2.16^bc^ | 82.32±2.23^ab^ |
|  | 400 | 63.33±1.00^bc^ | 81.81±1.63^a^ | 83.56±0.89^a^ |
|  | 500 | 60.67±1.25^bc^ | 67.25±0.82^c^ | 74.32±1.45^c^ |
|  | 600 | 50.03±1.50^d^ | 60.53±1.63^c^ | 62.65±1.11^d^ |
|  |  |  |  |  |
|  | CK | 84.17±1.25^a^ | 0.00±0.00^e^ | 84.17±1.25^b^ |
|  | 50 | 81.67±2.05^ab^ | 22.36±1.52^d^ | 83.66±2.43^b^ |
|  | 100 | 86.67±0.47^a^ | 25.11±2.34^d^ | 91.26±0.25^a^ |
| NaCl:Na_2_SO_4_=1:1 | 200 | 73.33±1.32^bc^ | 37.52±1.63^c^ | 80.47±2.25^b^ |
|  | 300 | 77.5±0.81^bc^ | 72.35±0.35^bc^ | 90.14±1.10^ab^ |
|  | 400 | 72.50±1.32^bc^ | 78.42±1.25^b^ | 91.21±0.41^a^ |
|  | 500 | 70.21±1.78^bc^ | 83.28±1.56^a^ | 77.01±3.12^c^ |
|  | 600 | 48.33±1.24^d^ | 88.05±0.94^a^ | 64.15±1.11^d^ |
|  |  |  |  |  |
|  | CK | 84.17±1.25^a^ | 0.00±0.00^e^ | 84.17±1.25^ab^ |
|  | 50 | 85.83±1.25^a^ | 23.53±0.47^d^ | 88.66±2.35^a^ |
|  | 100 | 76.67±0.94^ab^ | 53.57±0.82^c^ | 82.36±1.14^ab^ |
| NaHCO_3_:Na_2_CO_3_=1:1 | 200 | 72.50±1.63^ab^ | 60.62±1.25^b^ | 83.66±2.31^ab^ |
|  | 300 | 56.67±1.70^bc^ | 78.85±1.24^a^ | 64.35±1.48^c^ |
|  | 400 | 50.33±0.82^bc^ | 72.53±2.74^a^ | 61.56±0.42^c^ |
|  | 500 | 47.50±0.90^bc^ | 76.12±2.82^a^ | 51.11±0.98^d^ |
|  | 600 | 12.50±3.98^d^ | 65.53±1.63^ab^ | 40.25±2.51^e^ |

Note: Data represented mean±standard error (SE). Different superscript letters (a, b, c, d, e) in the columns indicated significant differences between means (*p*<0.05). Gs, Gr and Gt represented Germination percentage, Recovery germination percentage, and Total germination percentage.

**Supplementary Table S2.** Effect of the treatments of the mixed salt-alkali stress with different proportion on the seed germination of *H. caspica*

| **Different treatment** | **Salt concentration**  **(mmol/L)** | **G_s_ (A/C)*100** | **G_r_ [B/(C-A)]*100** | **G_t_ [(A+B)/C]*100** |
| --- | --- | --- | --- | --- |
|  | CK | 84.17±1.25^a^ | 0.00±0.00^c^ | 84.17±1.25^a^ |
|  | 50 | 83.83±1.25^a^ | 55.32±0.47^bc^ | 92.77±1.56^a^ |
|  | 100 | 69.17±1.24^b^ | 48.21±0.82^ab^ | 84.03±2.12^ab^ |
| NaCl:NaHCO_3_=1:1 | 200 | 52.20±1.20^bc^ | 71.67±2.05^a^ | 86.45±0.25^ab^ |
|  | 300 | 51.67±0.25^bc^ | 75.86±1.25^a^ | 88.33±1.23^ab^ |
|  | 400 | 50.00±1.20^bc^ | 76.86±2.30^a^ | 88.43±3.21^ab^ |
|  | 500 | 5.83±2.47^d^ | 76.92±1.63^a^ | 72.26±1.10^c^ |
|  | 600 | 4.17±2.45^d^ | 78.26±0.86^a^ | 68.38±2.10^c^ |
|  |  |  |  |  |
|  | CK | 84.17±1.25^a^ | 0.00±0.00^d^ | 84.17±1.25^a^ |
|  | 50 | 81.67±1.70^ab^ | 22.72±0.47^c^ | 85.83±0.25^ab^ |
|  | 100 | 78.33±1.87^ab^ | 26.92±1.82^c^ | 84.38±1.52^ab^ |
| NaCl:NaHCO_3_=1:3 | 200 | 67.50±3.56^bc^ | 35.10±1.12^bc^ | 78.90±0.25^ab^ |
|  | 300 | 67.50±1.25^bc^ | 32.45±2.25^bc^ | 82.98±2.74^ab^ |
|  | 400 | 40.83±2.05^d^ | 60.00±1.85^a^ | 78.04±1.41^c^ |
|  | 500 | 0.83±0.47^e^ | 58.92±0.87^ab^ | 59.26±1.48^d^ |
|  | 600 | 0.00±0.00^f^ | 52.32±1.46^ab^ | 52.32±1.46^d^ |
|  |  |  |  |  |
|  | CK | 84.17±1.25^a^ | 0.00±0.00^f^ | 84.17±1.25^ab^ |
|  | 50 | 85.83±1.69^a^ | 23.53±0.47^e^ | 89.16±1.03^a^ |
|  | 100 | 77.50±0.87^b^ | 33.33±0.82^d^ | 85.00±0.85^ab^ |
| NaCl:NaHCO_3_=3:1 | 200 | 67.50±0.56^cd^ | 50.16±1.26^c^ | 83.80±0.82^ab^ |
|  | 300 | 66.67±0.94^c^ | 66.66±0.29^bc^ | 88.89±0.74^ab^ |
|  | 400 | 65.50±2.44^cd^ | 67.66±0.82^bc^ | 88.84±1.41^b^ |
|  | 500 | 48.33±1.89^d^ | 77.42±1.62^a^ | 88.33±1.48^bc^ |
|  | 600 | 37.50±0.82^de^ | 69.33±1.70^ab^ | 80.83±1.12^bc^ |

Note: Data represented mean±standard error (SE). Different superscript letters (a, b, c, d, e, f) in the columns indicated significant differences between means (*p*<0.05). Gs, Gr and Gt represented Germination percentage, Recovery germination percentage, and Total germination percentage.

.

**Supplementary Table S3.** Relative salt damage percentage and salt tolerance level of different salt-alkali treatments on the seed germination of *H. caspica.*

| **Salt type** | **Salt concentration** | **RSD** | **Salt tolerance level** |
| --- | --- | --- | --- |
|  | 50 | 7.88±0.38^bc^ | One-level |
|  | 100 | -4.17±1.96^d^ | One-level |
| NaCl | 200 | 1.75±2.03^c^ | One-level |
|  | 300 | 7.77±1.58^bc^ | One-level |
|  | 400 | 2.98±0.04^c^ | One-level |
|  | 500 | 11.72±1.48^b^ | One-level |
|  | 600 | 21.57±2.34^a^ | Three-level |
|  |  |  |  |
|  | 50 | 6.93±0.45^d^ | One-level |
|  | 100 | 17.78±2.75^c^ | One-level |
| NaHCO_3_ | 200 | 17.79±0.69^c^ | One-level |
|  | 300 | 18.50±2.90^c^ | One-level |
|  | 400 | 26.69±2.29^b^ | Three-level |
|  | 500 | 34.51±1.98^ab^ | Three-level |
|  | 600 | 39.50±1.49^a^ | Three-level |
|  |  |  |  |
|  | 50 | 2.70±2.96^c^ | One-level |
|  | 100 | -3.06±0.85^d^ | One-level |
| NaCl:Na_2_SO_4_=1:1 | 200 | 12.82±1.13^b^ | One-level |
|  | 300 | 13.80±0.79^b^ | One-level |
|  | 400 | 11.75±1.29^b^ | One-level |
|  | 500 | 16.81±0.28^b^ | One-level |
|  | 600 | 42.42±1.53^a^ | Five-level |
|  |  |  |  |
|  | 50 | 2.11±1.25^e^ | One-level |
|  | 100 | 8.68±2.07^d^ | One-level |
| NaHCO_3_:Na_2_CO_3_=1:1 | 200 | 13.62±2.52^c^ | One-level |
|  | 300 | 32.39±2.56^bc^ | Three-level |
|  | 400 | 34.59±0.95^bc^ | Three-level |
|  | 500 | 43.42±1.41^b^ | Five-level |
|  | 600 | 85.19±0.71^a^ | Nine-level |

Note: Data represented mean±standard error (SE). Different superscript letters (a, b, c, d, e) in the columns indicated significant differences between means (*p*<0.05). RSD represented Relative salt damage percentage. The evaluation of salt tolerance on the *H. caspica* seed germination was followed the criteria outlined in Wang et al.^39^.

**Supplementary Table S4.** Relative salt damage percentage and salt tolerance level under the treatments of the mixed salt-alkali stress with different proportion on the seed germination of *H. caspica*.

| **Salt type** | **Salt concentration** | **RSD** | **Salt tolerance level** |
| --- | --- | --- | --- |
|  | 50 | 2.79±1.59^d^ | One-level |
|  | 100 | 17.67±1.41^c^ | One-level |
| NaCl:NaHCO_3_=1:1 | 200 | 37.51±1.27^b^ | Three-level |
|  | 300 | 38.46±1.79^b^ | Three-level |
|  | 400 | 40.45±1.44^b^ | Five-level |
|  | 500 | 93.01±0.56^a^ | Nine-level |
|  | 600 | 95.10±0.42^a^ | Nine-level |
|  |  |  |  |
|  | 50 | 4.79±1.61^e^ | One-level |
|  | 100 | 6.96±1.22^e^ | One-level |
| NaCl:NaHCO_3_=1:3 | 200 | 19.73±3.51^d^ | One-level |
|  | 300 | 38.64±0.79^c^ | Three-level |
|  | 400 | 51.63±1.47^b^ | Three-level |
|  | 500 | 99.05±0.45^a^ | Nine-level |
|  | 600 | 100.00±0.00^a^ | Nine-level |
|  |  |  |  |
|  | 50 | -0.33±2.97^e^ | One-level |
|  | 100 | 7.88±0.39^d^ | One-level |
| NaCl:NaHCO_3_=3:1 | 200 | 18.85±1.51^c^ | One-level |
|  | 300 | 19.18±1.93^c^ | One-level |
|  | 400 | 20.58±2.45^c^ | Three-level |
|  | 500 | 42.54±1.83^b^ | Five-level |
|  | 600 | 55.36±1.09^a^ | Five-level |

Note: Data represented mean±standard error (SE). Difference superscript letters (a, b, c, d, e) in the columns indicated significant differences between means (*p*<0.05). RSD represented Relative salt damage percentage. The evaluation of salt tolerance on the *H. caspica* seed germination was followed the criteria outlined in Wang et al.^39^.
